# A Novel Oncogenic Driver in a Lung Adenocarcinoma Patient Harboring an *EGFR*-KDD and Response to Afatinib

**DOI:** 10.3389/fonc.2020.00867

**Published:** 2020-06-16

**Authors:** Dong Chen, Xing-liang Li, Biao Wu, Xiao-bin Zheng, Wen-xian Wang, Hua-fei Chen, Yi-yu Dong, Chun-wei Xu, Mei-yu Fang

**Affiliations:** ^1^Department of Pathology, Zhejiang Rongjun Hospital, Jiaxing, China; ^2^Department of Tumor Molecular Laboratory, Zhejiang Rongjun Hospital, Jiaxing, China; ^3^Department of Medical Thoracic Oncology, Fujian Cancer Hospital, Fujian Medical University Cancer Hospital, Fuzhou, China; ^4^Department of Chemotherapy, Zhejiang Cancer Hospital, Hangzhou, China; ^5^Department of Thoracic Disease Diagnosis and Treatment Center, Zhejiang Rongjun Hospital, Jiaxing, China; ^6^Department of Pathology, Fujian Cancer Hospital, Fujian Medical University Cancer Hospital, Fuzhou, China

**Keywords:** non-small cell lung cancer, epidermal growth factor receptor, kinase domain, next-generation sequencing, afatinib

## Abstract

**Introduction:** Oncogenic mutations in the epidermal growth factor receptor (*EGFR*) occur frequently in patients with lung cancer. These mutations may serve as critical predictive biomarkers in patients with non-small cell lung cancer (NSCLC). Among them, *EGFR* exon 18–25 kinase domain duplication (*EGFR*-KDD) mutations have been identified as a novel *EGFR* gene subtype in NSCLC.

**Case Presentation:** We reported a rare case of a 59-year-old male diagnosed with adenocarcinoma. A biopsy revealed an *EGFR*-KDD identified by the next generation sequencing (NGS). Effective treatment outcome has been observed after administration with afatinib.

**Conclusion:** This case highlights that comprehensive NGS technique is valuable in detecting novel genetic mutations in tumors.

## Introduction

Non-small cell lung cancer (NSCLC) is the leading cause of cancer-related death, with adenocarcinoma being one of the most common forms (1). Epidermal growth factor receptor (*EGFR*) mutations could be detected in 30–60% of Asian patients and 10–20% of Caucasian patients with lung cancer (2). Being as a driver oncogene, double-blinded randomized clinical trials have indicated that application of EGFR tyrosine kinase inhibitors (TKIs) are effective against NSCLC cases harboring *EGFR* mutations (3).

*EGFR* mutations most commonly occur in exon 19 or exon 21 within the EGFR tyrosine kinase domain. The rare *EGFR* mutations are usually not detected by the first-generation testing techniques. However, advanced precise detection techniques (e.g., next generation sequencing, NGS) enable the discovery of more rare *EGFR* mutations, including exons 18–25 kinase domain duplications (KDDs) (4). Herein, we reported a first case of an oncogenic EGFR-KDD in lung adenocarcinoma who were responsive to treatment with afatinib in Chinese populations.

## Case Presentation

**Figure 1:**
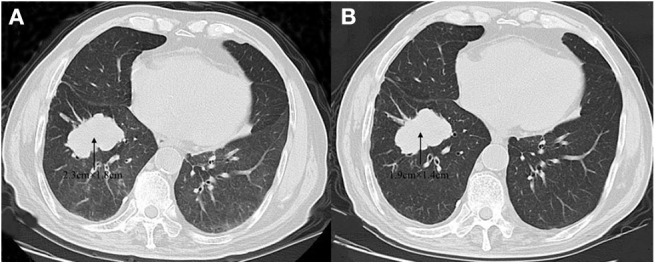
Computed tomography (CT) scans before **(A)** and after **(B)** afatinib therapy.

**Figure 2:**
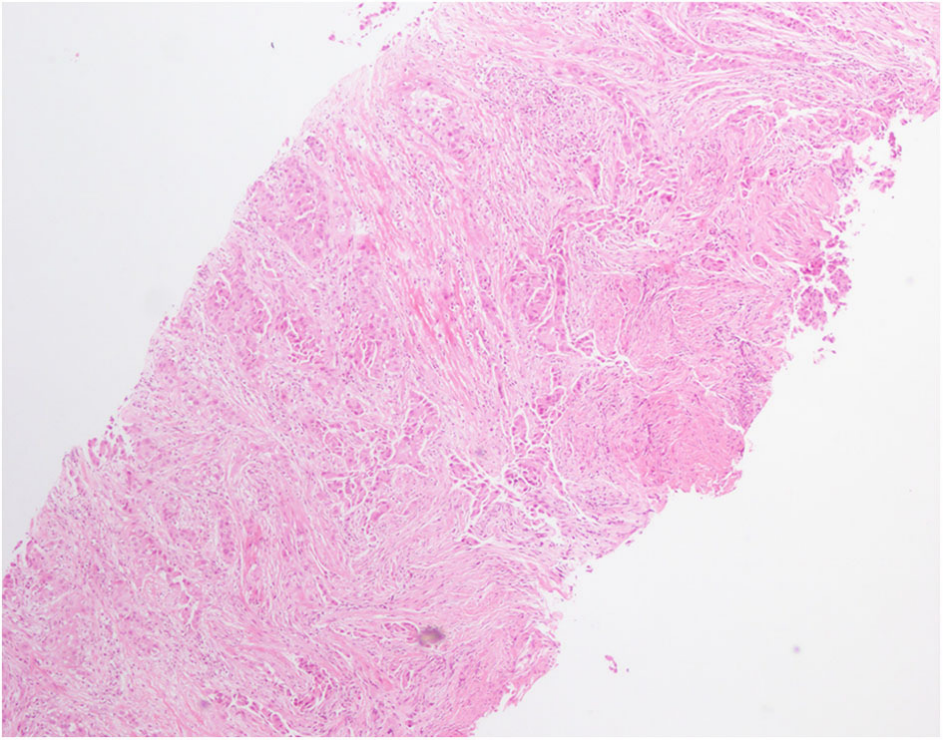
Hematoxylin and eosin (H&E) staining of lung biopsy showed a typical morphology for adenocarcinoma cells (H&E × 100).

**Figure 3:**
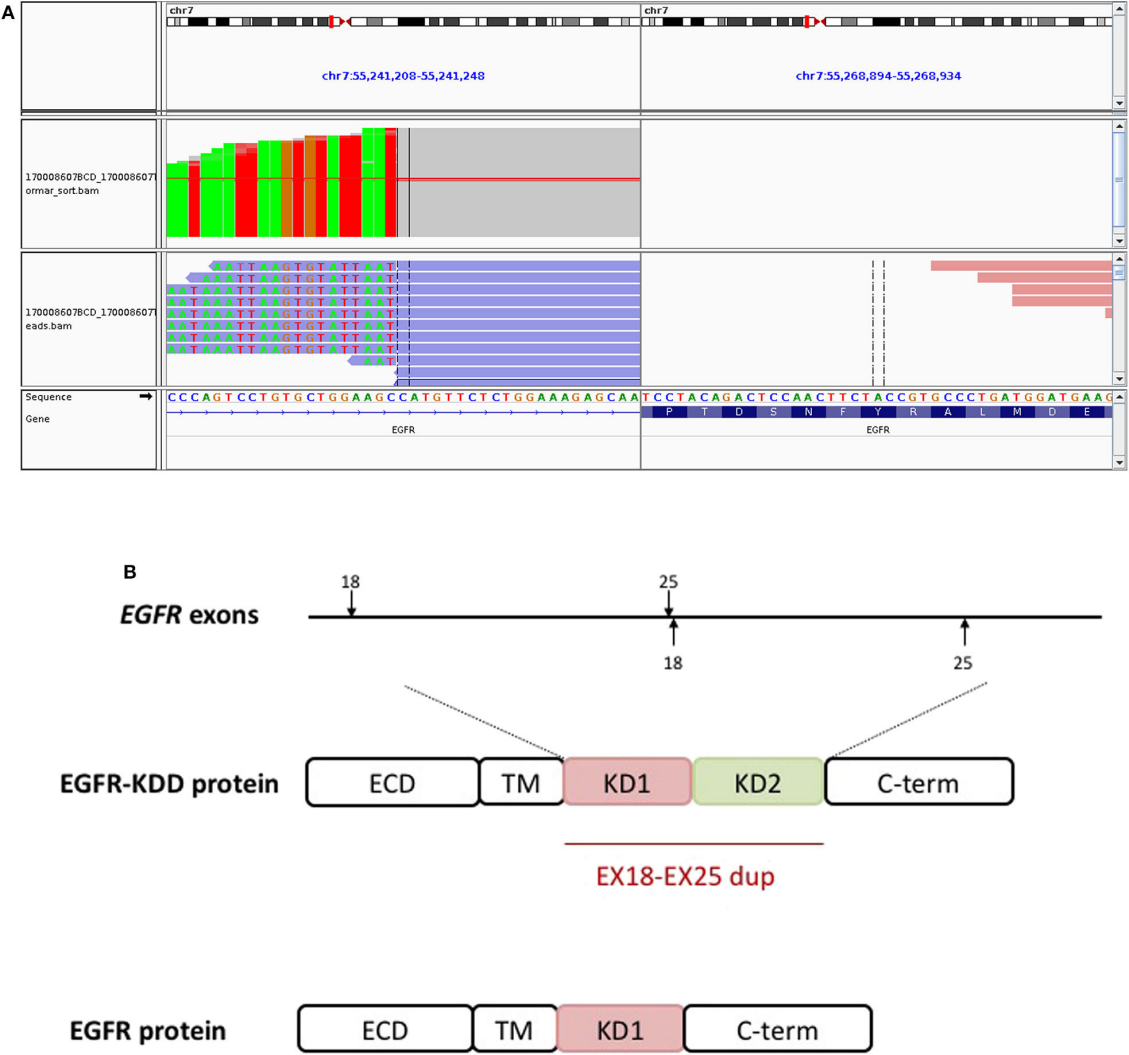
The *EGFR*-KDD is an oncogenic *EGFR* alteration. **(A)** The Integrative Genomics Viewer snapshot of paired NGS reads of tumor and matched blood. **(B)** Schematic representation of *EGFR*-KDD depicting the genetic and protein domain structures. ECD, extracellular domain; TM, transmembrane domain; KD1, first kinase domain; KD2, second kinase domain; C-term, carboxyl terminus. Blue, *EGFR* exons 18–25 #1; orange, *EGFR* exons 18–25 #2.

A 59-year-old male was referred to our hospital for detection of a right lung mass on physical examination. He had no history of pulmonary disease or smoking. A mass (2.3 × 1.8 cm) in the right lower lung was observed by computed tomography (CT) scan (Figure 1A). F-18 fluorodeoxyglucose hypermetabolic speckles in fourth vertebral body; no hypermetabolic lesions were demonstrated in other sites, and a MRI of the brain or CT of the head with IV contrast was not performed. We detected a typical morphology for adenocarcinoma cells by hematoxylin and eosin (H&E) staining (Figure 2). Immunohistochemical staining showed positive for the expression of NapsinA, TTF-1, and CK7. The patient was classified as stage IV (T_1_N_0_M_1_), in accordance to the 7th edition of TNM staging. The ARMS assay, the first-generation sequencing technique, revealed wild-type for sensitive *EGFR* mutations, including *EGFR* 18-21, and negative for *ALK* rearrangement or *ROS1* rearrangement. Then, a NGS analysis of the tumor biopsy identified a *EGFR*-KDD mutation (copy number 2.0) accompanied *TP53* p.R282W (frequency 13.0%) and *CTNNB1* p.S37Y (frequency 5.1%) in the tumor (Geneplus, Beijing, China) (Figure 3), and PD-L1 staining was not done. Therefore, he underwent oral afatinib treatment (30 mg qd). Afterwards, the patient showed a stable tumor response to afatinib (1.9 × 1.4 cm) (Figure 1B). Besides, there were no adverse events, including gastrointestinal reactions, hepatic and renal function, and cardiac damage. Currently, the disease is stable and treatment with afatinib continues for 10 months.

## Discussion

Oncogenic *EGFR* mutations are detected in 30–60% of Asian patients and 10–20% of Caucasian patients. Such mutations are most detected as small in-frame deletions in exon 19 or point mutations in exon 21. Uncommon *EGFR* alterations, including rare point mutations and gene rearrangements, have also been reported previously (5).

*EGFR*-KDDs could activate EGFR signaling by forming an intra-molecule dimer (6). *EGFR*-KDD of exons 18–25, firstly discovered in a glioblastoma (7), has been recognized as a driver gene in lung cancer. In 2015, Baik et al. (8) first reported an *EGFR*-KDD in a female patient with a bronchoalveolar carcinoma and responsive to the first-generation EGFR-TKIs, including erlotinib and gefitinib. Another case reported a male patient with lung adenocarcinoma and an *EGFR*-KDD mutation detected with NGS, who had a preferable anti-tumor response to afatinib, a second-generation EGFR-TKI (6). Zhu et al. (9) reported the first case involving the presence of oncogenic *EGFR*-KDD in China, who had stable disease to treatment with an EGFR-TKI icotinib. Another case report of a prolonged multi-year response to gefitinib and then erlotinib has been described for advanced *EGFR*-KDD mutated lung adenocarcinoma (8). Therefore, it seems these *EGFR* variants are sensitive to first- and second- generation EGFR-TKIs. Consistently, *in vitro* study showed that *EGFR*-KDD is constitutively active, and computational modeling provides potential mechanistic support for its auto-activation (9). Herein, we for the first time detected EGFR-KDD in a Chinese patient who achieved sustained anti-tumor responses from treatment with afatinib.

Polymerase chain reaction (PCR) is frequently applied for detection of common *EGFR* variants in NSCLC patients, which is unable to identify some rare types of *EGFR* alterations (10). By contrast, NGS allows for multiplex testing and enables the detection of known as well as uncommon genomic events, as reported in this case (11). Thus, clinical treatment should improve with clinical diagnostics for multiple gene testing to provide personalized cancer therapy.

In summary, the present case increases the evidence supporting afatinib treatment of NSCLC patients harboring *EGFR*-KDD variants. The NGS assay provides a useful way to identify rare and uncommon *EGFR* gene mutations in NSCLC patients.

## Ethics Statement

This study was approved by the Ethic Committee of Zhejiang Rongjun Hospital. The patient provided written informed consent for the publication of this case report.

## Author Contributions

DC, XL, and BW conceptualized and designed the entire study. XZ, HC, and MF carried out patient clinical management and sample collection. WW analyzed the data. WW, YD, and CX wrote the manuscript. WW and CX revised the manuscript. All authors read and approved the final version of manuscript for submission.

## Conflict of Interest

The authors declare that the research was conducted in the absence of any commercial or financial relationships that could be construed as a potential conflict of interest.
